# Factors Associated With Prescription Opioid Use Among Community-Dwelling Older Adults

**DOI:** 10.1093/geroni/igaa057.669

**Published:** 2020-12-16

**Authors:** Carina D’Aiuto, Helen-Maria Vasiliadis

**Affiliations:** 1 Université de Sherbrooke, Longueuil, Quebec, Canada; 2 University of Sherbrooke, Sherbrooke, Quebec, Canada

## Abstract

Opioid use is a growing concern in North America, particularly among older adults. Despite the opioid crisis and the aging population, few studies have evaluated the factors associated with opioid use among older adults. Our sample includes 1657 people aged ≥65 years recruited in primary care clinics from 2011 to 2013 in the Montérégie region of Québec and participating in the “Étude sur la Santé des Aînés” ESA-Services study, a longitudinal study on aging and health service use. The presence of chronic diseases was identified through self-reported health survey data linked to health administrative data. Opioid prescriptions were identified using the provincial pharmaceutical drug registry for those covered under the public drug insurance plan. Logistic regression analyses were conducted to examine the factors associated with opioid use over a 4-year period. 31.9% of participants used opioids. Factors associated with opioid use included: female sex (OR=1.24, 95%CI: 1.01-1.53), annual household income of <$25,000 (OR=1.25, 95%CI: 1.01-1.55), level of social support (OR=0.85, 95%CI: 0.73-0.99), and presence of pain/discomfort (OR=1.66, 95%CI: 1.34-2.04). Further, participants with ≥3 chronic physical conditions also reporting anxiety and/or depression were 3.63 (95%CI: 1.83-7.18) times more likely to use an opioid than those with 0-2 chronic physical conditions and no common mental disorder. Moreover, those with moderate, high, and very high psychological distress were more likely to use an opioid than those with a low psychological distress. Our findings suggest that, among other factors, physical and psychiatric multimorbidity is strongly associated with prescription opioid use in older adults.

